# Determinants of pubertal progression and final height in premature pubarche

**DOI:** 10.1007/s40618-026-02957-6

**Published:** 2026-06-24

**Authors:** Didem Helvacioglu, Aylin Tugba Canbaz, Mehmet Eltan, Feride Cosar, Serap Turan, Abdullah Bereket, Tulay Guran

**Affiliations:** Department of Pediatric Endocrinology, Marmara University, School of Medicine, Istanbul, 34854 Turkey

**Keywords:** Premature adrenarche, Final height, Bone age, DHEA-S, Premature pubarche, Precocious puberty

## Abstract

**Background:**

Premature pubarche (PP), defined as the early onset of pubic hair and often associated with mild androgen excess, is a common reason for referral to a pediatric endocrinologist. A small proportion of cases may progress to central precocious puberty (CPP). Data on the factors determining pubertal tempo and associated growth outcomes in these individuals are limited.

**Objective:**

To identify predictors of pubertal progression and final height (FH) in girls with PP and to evaluate growth outcomes in those progressing to CPP.

**Design-methods:**

We retrospectively analyzed 88 girls with PP who attained FH; 13 progressed to CPP (PP-to-CPP). Auxological data, bone age (BA), pubertal development, and plasma androgen profiles were evaluated.

**Results:**

At presentation, corrected height SDS (CH-SDS) in girls with PP correlated with BA-SDS and BA/chronological age (BA/CA) ratio (p=0.03 and p=0.007). Corrected FH-SDS was positively associated with baseline CH-SDS in both PP and PP-to-CPP groups (p<0.001 and p=0.0002). Compared with PP, the PP-to-CPP group had higher baseline CH-SDS, BA-SDS and BA/CA ratio (all p=0.02).

Baseline CH-SDS was the strongest independent predictor of corrected FH-SDS (p<0.001), while BMI-SDS showed a negative association (p=0.003). Baseline dehydroepiandrosterone-sulfate (DHEA-S) was not associated with CH-SDS, BA advancement, progression to CPP, or corrected FH-SDS in either group. Receiver operating characteristic (ROC) analyses for progression to CPP identified a BA/CA cutoff of 1.2; and a CH-SDS cutoff of 1.8 (p=0.01).

**Conclusion:**

Growth relative to genetic potential and skeletal maturation at presentation were the strongest predictors of progression to CPP and FH outcomes in girls with PP. Although DHEA-S reflects adrenal maturation and early androgen exposure, baseline DHEA-S concentrations were not associated with progression to CPP or long-term growth outcomes in our cohort.

**Supplementary Information:**

The online version contains supplementary material available at https://doi.org/10.1007/s40618-026-02957-6.

## Introduction

Premature pubarche (PP) is defined as the appearance of pubic and/or axillary hair before 8 years of age in girls and 9 years in boys, without other signs of central puberty. Over 90% of children presenting with PP have premature adrenarche (PA)—a slowly progressive condition with mild adrenal androgen excess, particularly dehydroepiandrosterone (DHEA) and dehydroepiandrosterone sulfate (DHEA-S) [1]. Although a normal pubertal variant, it remains a diagnosis of exclusion. The differential diagnosis includes central precocious puberty (CPP), non-classic congenital adrenal hyperplasia (CAH), virilizing adrenal or ovarian tumors, and exogenous androgen exposure. Particularly in girls, PP is the most common cause of referral for precocious puberty in pediatric endocrinology clinics [2].

Advanced bone age (BA) and accelerated growth, frequently observed in PP, raise concerns regarding progression to CPP and potential compromise of final height (FH); accordingly, extensive laboratory evaluations are often performed to address these concerns and exclude underlying pathology [3].

However, available longitudinal data indicate that, in most children with idiopathic PP, FH is preserved and remains close to genetic potential [4–12] (Supplementary Material). In fact, progression from PP to CPP has been reported in approximately 13% of cases; however, predictors of pubertal tempo and long-term growth outcomes, particularly among those who progress to CPP, remain incompletely defined [13].

DHEA-S is considered the principal biochemical marker of adrenarche because of its adrenal origin, relatively high circulating concentrations, and age-related increase during adrenal maturation. Adrenal androgen secretion, particularly DHEA-S, has been associated with a range of cross-sectional and short-term follow-up features of PP. Higher DHEA-S concentrations have been reported in children with PP who later progress to CPP, in those with BA advancement even in the absence of pubertal progression, and in children with obesity or born small for gestational age (SGA) [10, 14–17]. Although DHEA-S levels are typically elevated at presentation and tend to decline over time, their relationship with long-term growth outcomes, particularly FH, remains unclear [16]. Notably, in our previous prospective study, circulating adrenal androgens (including DHEA-S and androstenedione) were not associated with height SDS, corrected height (CH), or growth velocity during short- to mid-term follow-up in girls with isolated PP [18] .

This study aimed to evaluate the relationship between baseline adrenal androgens (particularly DHEA-S), skeletal maturation and FH outcomes in girls with PP, and to assess determinants of progression to CPP.

## Materials and methods

### Study setting and patients

This retrospective study included 88 girls diagnosed with PP who were followed at a tertiary pediatric endocrinology center between 1999 and 2025 and who reached FH during follow-up. Seventy-five girls did not progress to CPP (PP group) (85%), whereas 13 progressed to CPP (15%) according to established diagnostic criteria [19, 20] and received gonadotropin-releasing hormone agonist (GnRHa) therapy (PP-to-CPP group). The mean duration of treatment was 2.5±0.7 years.

Data collected from medical records included age at presentation and at onset of pubic/axillary hair, gestational age, parental heights and mid-parental height, serial anthropometry, BA, and serum hormone concentrations (DHEA-S, androstenedione, total testosterone, and 17-hydroxyprogesterone) at diagnosis and during follow-up. At FH, BMI, DHEA-S, age at menarche, menstrual pattern, and hirsutism status were recorded.

### Clinical and biochemical evaluation

Height was measured using a wall-mounted Harpenden stadiometer (Holtain Ltd, Crymych, UK; 0.1 cm precision) and weight using an electronic scale (0.1 kg precision). BMI was calculated as weight/height^2^ (kg/m^2^). All anthropometric variables were expressed as standard deviation scores (SDS) using Turkish reference standards [21, 22]. Mid-parental (target) height for girls was calculated as [(father’s height – 13 cm) + mother’s height]/2. Corrected height (CH) SDS was calculated as height SDS minus mid-parental height SDS (CH-SDS= height-SDS − MPH-SDS).

Final height (FH) was defined as the height attained when growth velocity was <1 cm/year and/or bone age (BA) reached 14 years in girls. Pubertal development was assessed using the Tanner classification [23]. BA was calculated as the mean of assessments performed independently by three investigators (DH, ATC, TG) using the Greulich-Pyle method, BA-SDS and predicted adult height (PAH) were calculated based on the Greulich-Pyle atlas [24]. Inter-observer variability was less than or equal to 6 months, and the mean of the measurements was used for analysis. DHEA-S levels were analyzed using commercial kits based on a nonisotopic automated chemiluminescence immunoassay system (ADVIA Centaur, Siemens Diagnostics, Erlangen, Germany). The lower and upper limits of detection for the assay were 3 and 1500 µg/dL (81 and 4065 nmol/L), respectively. DHEA-S concentrations above 1084 nmol/L (40 µg/dL) were considered indicative of adrenal activation. In addition, age- and sex-adjusted DHEA-S SDSs were calculated [25]. In all patients, both classic and non-classic forms of CAH, CPP, androgen-secreting tumors and some other rare causes of androgen excess, were excluded at initial evaluation through clinical, biochemical, and imaging assessments, using an algorithm we developed to establish the differential diagnosis and follow-up plan in girls presenting with premature pubarche [26, 27].

### Statistical analysis

Statistical analyses were performed using GraphPad Prism version 5.0 (GraphPad Software, San Diego, CA, USA). Normality was assessed using the Shapiro–Wilk test. Continuous variables are presented as mean±SD or median (IQR), as appropriate, and categorical variables as n (%). Between-group comparisons were performed using Student’s t-test for normally distributed data and the Mann–Whitney U test for non-normally distributed data. Correlations were assessed using Pearson’s or Spearman’s correlation coefficients, as appropriate. Receiver operating characteristic (ROC) curve analyses were performed to evaluate the ability of baseline BA/CA ratio and CH-SDS to predict progression to CPP; areas under the curve (AUCs) were calculated and optimal cut-offs were identified, with corresponding sensitivity and specificity reported. A two-sided p-value <0.05 was considered statistically significant.

### Ethics

This study was conducted in accordance with the principles of the Declaration of Helsinki. The protocol was approved by the Institutional Ethics Committee (Approval No: 09.2026.26-0430), and written informed consent was obtained from legal guardians.

## Results

### Baseline clinical characteristics of PP and PP-to-CPP groups

Among 88 girls who reached FH, 75 (85%) had PP (age at onset 6.71±1.07 years), and 13 had PP-to-CPP (6.44±1.25 years). Gestational age, frequency of SGA, maternal age at menarche and pubic hair (PH) stages were similar in PP and PP-to-CPP groups (Table 1). Baseline age and anthropometric indices, including weight, height and BMI-SDS were comparable between groups (Table 2).

**Table 1:** Clinical characteristics of PP and PP-to-CPP groups

	PP (n=75)	PP-to-CPP (n=13)	*p*
SGA (n)	6/75	1/13	0.99
Age of menarche (yr)	11.76±0.95	12.02±0.91	0.08
Only pubic hair at presentation (n)	47/75	10/13	0.36
Only axillary hair at presentation (n)	7/75	-	0.59
Pubic and axillary hair at presentation (n)	21/75	3/13	0.99
MPH-SDS	-0.49±0.92	-0.64±1.12	0.62
Mother’s age of menarche (yr)	12.7±1.4	12.7±1.3	0.72

PP, premature pubarche; CPP, PP-to-CPP, progression from premature pubarche to central precocious puberty; SGA, small for gestational age; MPH, mid-parental height; SDS, standard deviation score

**Table 2:** Comparison of clinical and biochemical characteristics in PP and PP-to-CPP groups at baseline and final height

	1.Evaluation			Final evaluation		
	**PP**	PP-to-CPP	p	PP	PP-to-CPP	p
Age (yr)	7.56±1.23	7.41±0.89	0.59	15.06±1.63	15.89±2.11	0.19
Height SDS	0.70±1.08	1.26±1.20	0.11	-0.23±1.08	-0.02±1.10	0.56
CH-SDS	1.19±0.85	1.90±1.23	**0.02**	0.25±1.00	0.74±1.03	0.16
Height (cm)	127±9.79	129.5±4.75	0.45	161.7±6.37	164.1±6.68	0.23
Weight SDS	1.01±1.19	1.01±0.98	0.81	1.28±1.86	1.05±1.33	0.77
BMI-SDS	0.90±1.23	0.52±1.07	0.32	1.00±1.74	1.03±1.54	0.97
BA-SDS	1.06±1.30	2.16±1.33	**0.02**			
BA/CA	1.12±0.17	1.25±0.15	**0.02**			
PAH (cm)	163.1±6.46	163.5±11.45	0.53			
17OH-P (ng/mL)	0.86±0.53	0.91±0.59	0.98			
DHEA-S (mcg/dL)	85.1±52.2	83.9±33.6	0.71	287.3±93.11	327.2±211.0	0.51
DHEA-S SDS	2.07±1.94	1.21±1.37	0.16	1.13±1.28	0.92±1.89	0.72

PP, premature pubarche; PP-to-CPP, progression from premature pubarche to central precocious puberty; BA, bone age; CA, chronological age; BA/CA, bone age to chronological age ratio; BMI, body mass index; CH, height corrected for mid-parental height; DHEA-S, dehydroepiandrosterone sulfate; SDS, standard deviation score; 17OH-P, 17-hydroxyprogesterone

### Baseline hormone measurements and skeletal maturation of PP and PP-to-CPP groups

Baseline CH-SDS was higher in the PP-to-CPP group than in the PP group despite similar mean DHEA-S concentrations (Table 2).

At baseline, mean BA was 8.23±1.93 years (1.06±1.30 SDS) in PP and 9.01±1.43 years (2.16±1.33 SDS) in PP-to-CPP. Both BA-SDS and BA/CA ratio were higher in girls who progressed to CPP (p=0.02). However, PAH did not differ between PP cases who later progressed to CPP and those who did not (163.1±6.46 cm vs 163.5±11.5 cm, p=0.53) (Table 2).

In the PP group, CH-SDS correlated with BA/CA ratio (r=0.42, p=0.007) and BA-SDS (r=0.35, p=0.03). In contrast, baseline DHEA-S was not correlated with CH-SDS (r=0.26, p=0.11), BA-SDS (r=0.26, p=0.11), or BA/CA ratio (r=0.18, p=0.29). DHEA-S concentrations also did not differ between overweight/obese PP patients (BMI-SDS > +1; n=34) and those with normal BMI (BMI-SDS ≤ +1; n=41) (p=0.59).

ROC analyses were performed to evaluate the ability of baseline BA/CA ratio and CH-SDS to predict progression from PP-to-CPP. The BA/CA ratio yielded an AUC of 0.73; a cutoff of 1.2 provided 70% sensitivity and 73% specificity (p=0.012). Baseline CH-SDS yielded an AUC of 0.72; a cutoff of 1.8 achieved 80% sensitivity and 70% specificity (p=0.01).

### Longitudinal clinical characteristics of PP and PP-to-CPP Groups

**Fig. 1:**
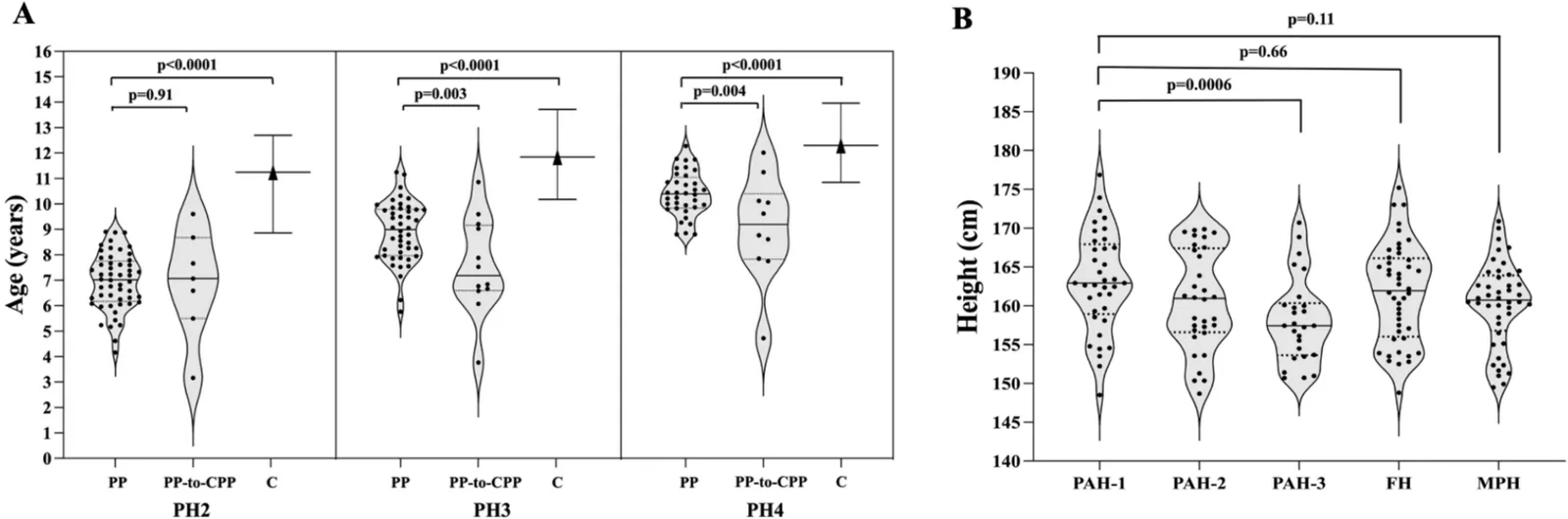
Progression of pubic hair development and changes in predicted adult height in girls with premature pubarche (PP), with premature pubarche progressing to central precocious puberty (PP-to-CPP). **A** Age at pubic hair stages (PH2–PH4) in PP, PP-to-CPP, and healthy control (C) groups. **B** Changes in predicted adult height across follow-up and comparison with final height and mid-parental height in PP group

The mean ages at PH2, PH3, and PH4 were 6.95±1.11, 8.90±1.18, and 10.39±0.88 years in the PP group and 6.90±2.13, 7.57±1.90, and 9.08±2.06 years in the PP-to-CPP group. Compared with healthy Turkish controls [28], girls in the PP group reached PH2, PH3, and PH4 at significantly younger ages (all p< 0.001). Girls in the PP-to-CPP group reached PH3 and PH4 at significantly younger ages than those in the PP group (respectively, p=0.003, p=0.004) (Fig. 1A).

In the PP group, PAH decreased across evaluations (163.1±6.46 cm at baseline, 160.7±6.47 cm at the second evaluation, and 158.2±5.53 cm at the third evaluation (p=0.006) (Table 3) (Fig. 1B). However, despite this decline in predicted height, FH remained consistent with genetic target height, suggesting that the observed reduction in PAH may reflect limitations of prediction models in the setting of advanced skeletal maturation rather than a true loss of growth potential.

**Table 3:** Growth and hormonal characteristics of girls with PP during follow-up and at final height

	1^st^ Evaluation	2^nd^ Evaluation	3^rd^ Evaluation	At final height	*p*
Time from baseline (years)		1.17±0.47	2.44±1.09	7.98±2.27	
Age (years)	7.56±1.23	8.48±0.99	9.76±1.47	15.06±1.63	
Height SDS	0.70±1.07	0.73±1.02	0.61±1.06	-0.23±1.08	**0.0001**
Weight SDS	1.13±1.19	1.04±1.16	0.87±1.19	1.23±1.86	0.5
BMI-SDS	0.91±1.23	0.89±1.27	0.63±1.40	1.01±1.74	0.35
BA-SDS	1.06±1.30	1.44±1.21	1.57±1.20		**0.01**
BA/CA	1.12±0.17	1.15±0.11	1.15±0.10		0.37
PAH (cm)	163.1±6.46	160.7±6.47	158.2±5.53		**0.006**
CH-SDS	1.19±0.85	1.30±0.81	1.31±0.89	0.25±0.99	**0.0001**
17OH-P (ng/mL)	0.86±0.53				
Testosterone (ng/dL)	0.11±0.09	0.16±0.17	0.35±0.54	39.83±32.13	
DHEA-S (mcg/dL)	85.1±52.2	111.5±52.02	134.5±48.02	287.3±93.11	
DHEA-S SDS	2.07±1.94	1.41±2.32	1.04±2.09	1.13±1.28	0.06

BA, bone age; CA, chronological age; BA/CA, bone age to chronological age ratio; BMI, body mass index; CH, height corrected for mid-parental height; DHEA-S, dehydroepiandrosterone sulphate; FH, final height; MPH, mid-parental height; SDS, standard deviation score; 17OH-P, 17-hydroxyprogesterone

During follow-up, in the PP group, BA and consequently height SDS were found to be overestimated relative to MPH-SDS, independent of DHEA-S and androgen concentrations; however, since the increase in height SDS paralleled the rise in CH-SDS, FH rechanneled in accordance with MPH (Table 3).

### Final height outcomes

Mean age at FH assessment was 15.06±1.63 years in girls with PP and 15.89±2.11 years in those who progressed to CPP. At FH, there were no significant differences in FH, weight, or BMI-SDS between PP and PP-to-CPP groups (Table 2).

In the PP group, FH-SDS did not differ from MPH-SDS (−0.23±1.08 vs −0.49±0.92; p=0.12), whereas in the PP-to-CPP group FH-SDS was higher than MPH-SDS (0.27±1.03 vs −0.76±1.19; p=0.04). Corrected FH-SDS did not differ between girls who progressed to CPP and those who did not (p=0.16).

In both groups, corrected FH-SDS was not correlated with baseline DHEA-S, BA/CA ratio, or BA-SDS. Corrected FH-SDS correlated positively with baseline CH-SDS in both groups (PP: r=0.55, p<0.001; PP-to-CPP: r=0.90, p=0.0002).

In multiple linear regression, baseline CH-SDS was the strongest independent predictor of corrected FH-SDS (p<0.001). BMI-SDS was negatively associated with corrected FH-SDS (p=0.003), whereas BA advancement and DHEA-S were not associated with corrected FH-SDS.

All girls with CPP received GnRHa therapy. Mean age at menarche was 11.76±0.95 years in PP and 12.02±0.91 years in the PP-to-CPP group, with no between-group difference (p=0.08) (Table 1). Among those reaching FH, hirsutism and menstrual irregularities were observed in 34.6% (25/75) and 17.1% (13/75) of the PP group, and in 38.5% (5/13) and 15.3% (2/13) of the PP-to-CPP group, respectively.

## Discussion

Premature pubarche (PP) is a common reason for referral to pediatric endocrine clinics, with reported prevalences ranging from approximately 4.3 -20% in girls [27, 29, 30]. Most of these children are healthy; yet extensive laboratory evaluations are often performed because of concerns about underlying pathology, progressive early puberty, and compromised FH. In our single-center cohort of 88 girls with PP followed to FH, representing one of the largest adult outcome series to date, FH closely matched genetic target height, confirming previous longitudinal reports of a favorable growth prognosis. The most striking finding of our study is that height relative to genetic target at the presentation emerged as the strongest independent predictor of adult height in both the PP and PP-to-CPP groups. By including a well-characterized subgroup of girls with PP who progressed to CPP, we demonstrate that advanced skeletal maturation at presentation—rather than adrenal androgen levels—was the primary predictor of hypothalamo-pituitary-gonadal activation. PP girls requiring GnRHa treatment achieved FH comparable to, and in some cases exceeding, their genetic target height. As all girls in the PP-to-CPP group received GnRHa therapy, FH outcomes in this subgroup should be interpreted in the context of treatment and cannot be assumed to reflect the natural course of CPP.

Ibáñez et al. demonstrated that FH in girls with PP closely correlated with height prognosis assessed at diagnosis and pubertal onset [5]. Similarly, Mejorado-Molano et al. showed that target height and height at diagnosis were the main predictors of adult stature in idiopathic PP [4]. Notably, DHEA-S was not retained as an independent determinant in their model despite its diagnostic relevance, a finding that is consistent with our results and further supports the concept that adrenal androgen elevation per se does not compromise intrinsic growth potential.

Current evidence suggests that elevation of DHEA-S concentration primarily reflects adrenal maturation and may be associated with earlier pubertal timing, rather than serving as a direct trigger of central activation [31]. Consistent with this, in our cohort, baseline DHEA-S concentrations and DHEA-S SDS did not differ between girls who remained PP and those who progressed to CPP. Moreover, DHEA-S SDS did not change significantly between the prepubertal and postpubertal periods in both groups. These findings suggest that baseline DHEA-S concentrations have limited value for predicting subsequent progression to CPP or long-term growth outcomes.

Previous studies have reported that the rate of progression to CPP in cases presenting with PP ranges between 9% and 13% [2, 13]. In our cohort, a comparable proportion of girls (14.6%) progressed from PP to CPP, supporting consistency of CPP risk estimates across cohorts. Girls who progressed to CPP had significantly higher height relative to genetic potential, reflected by a higher CH-SDS, and more advanced skeletal maturation. The clinical utility of CH-SDS is further highlighted by the observation that baseline height SDS did not differ significantly between girls who remained PP and those who progressed to CPP, whereas CH-SDS did. This suggests that assessment of height relative to genetic growth potential may be more informative than absolute height SDS alone when evaluating the risk of pubertal progression in girls with PP. This “taller-than-target height” phenotype is consistent with observations from large idiopathic CPP cohorts [32]. ROC analyses further showed that baseline BA/CA ≥ 1.2 and CH-SDS ≥ 1.8 provide clinically useful thresholds for identifying a higher-risk subgroup among PP girls progressing to CPP. However, the association between advanced bone age and progression to CPP should be interpreted with caution. Advanced skeletal maturation may reflect increased estrogen exposure accompanying activation of the hypothalamic-pituitary-gonadal axis rather than representing an independent driver of pubertal progression. Our study demonstrated an approximately 5 cm reduction in PAH across serial evaluations in girls with PP, which likely reflects dynamic recalibration of height prediction rather than true loss of growth potential. Similar auxological patterns were described by Ibáñez et al. [5], who observed that early tall stature and advanced BA in girls with PP were followed by normalization of growth tempo, resulting in FH consistent with genetic target despite initial PAH overestimation. In our cohort, the elevated baseline BA/CA ratio supports the interpretation that accelerated skeletal maturation contributed to downward adjustment of PAH estimates. This view is reinforced by recent data from Kanniainen et al. [33], showing that PAH may remain within the normal range in children with PP despite advanced BA, highlighting inherent limitations of prediction models in the context of early skeletal advancement.

Long-term hyperandrogenic manifestations such as hirsutism represent clinically relevant outcomes of PP. Hirsutism occurred in 33.7% (30/88) of girls with PP, a prevalence comparable to that reported in adolescent follow-up series. Miller et al. described hirsutism in 37.8% of adolescents with a history of benign PP, closely aligning with our findings [34]. However, baseline DHEA-S SDS at diagnosis did not differ between girls with and without subsequent hirsutism, suggesting limited prognostic value of initial adrenal androgen levels for later clinical hyperandrogenism. Consistent with this, Hawkins et al. reported that, beyond excluding nonclassical CAH, androgen-producing tumors and some more rare pathologies, baseline adrenal steroid profiles did not reliably predict which girls with PP would later develop hirsutism or oligomenorrhea [35].

Several limitations should be considered when interpreting our findings. The retrospective design may have introduced inherent limitations related to standardization of follow-up assessments. In addition, the study spans more than two decades, during which laboratory assays and analytical methods may have evolved. Although DHEA-S measurements were interpreted using age- and sex-adjusted reference standards, the potential influence of methodological changes over time cannot be entirely excluded. Furthermore, although our cohort includes a relatively large number of girls with PP who reached FH only 13 progressed to CPP. Therefore, subgroup analyses involving the PP-to-CPP group and the ROC-derived thresholds for BA/CA ratio and CH-SDS should be interpreted with caution. These cut-off values should be considered exploratory and require validation in larger independent cohorts before being applied in routine clinical practice.

## Conclusion

In conclusion, PP, after exclusion of pathological androgen excess conditions at initial presentation (Fig. 1), appears to represent a tempo variant of growth and skeletal maturation that does not adversely affect adult height attainment. Height relative to genetic target at the presentation emerges as the strongest independent predictor of FH in PP. DHEA-S at presentation appears to have limited prognostic value for progression to CPP, attainment of FH, and later hyperandrogenic manifestations. However, DHEA-S remains an important marker of adrenal maturation and may play a role in the early phenotypic expression of PP, despite its lack of association with subsequent pubertal progression and growth outcomes. The inverse association between obesity and FH further underscores the importance of weight status during follow-up. Clinically, our data suggest that routine serial bone age monitoring is not required in all PP cases; bone age appears most informative at presentation, where advanced skeletal maturation identifies a subgroup at increased risk for progression to precocious puberty (Fig. 1). Therefore, girls with isolated idiopathic PP can be effectively followed using clinical and anthropometric assessments alone, while the potential long-term metabolic and reproductive risks of this condition should be clarified through extended longitudinal follow-up studies.

## Data availability

The data that support the findings of this study are not publicly available due to patient privacy considerations but are available from the corresponding author upon reasonable request.

## Supplementary Information

Below is the link to the electronic supplementary material.

Supplementary file 1.
