# Supplementary material for: Determinants of pubertal progression and final height in premature pubarche
Source: J Endocrinol Invest. 2026 Jun 24;49(10):2795–802. doi: 10.1007/s40618-026-02957-6 (PMC13627192; doi:10.1007/s40618-026-02957-6)
Supplement: Supplementary file 1 [file 40618_2026_2957_MOESM1_ESM.docx]

**Supplementary Material.** Final height outcomes relative to mid-parental height reported in previous studies of girls with PP.

| **Reference** | **Final Height** | **Mid-parental height** | **Number of patients (n)** | ***p* value** |
| --- | --- | --- | --- | --- |
| **Ibanez et al, 1992 [5]** | 160±5.3 cm | 155.6±5.3 cm | 38 | 0.0005 |
| **Pere et al, 1995 [6]** | 0.22±1.07 SDS  (1.83-1.72) | 0.22±0.58 SDS | 13 | NA |
| **Ghizzoni et al, 2000 [7]** | 162.7 cm  (160.8-164.6) | 159.5 cm  (157.7-161.3) | 38 | * |
| **Ibanez et al, 2006 [8]** | 160.7, 0.4 SDS | 158.5, 0.3 SDS | 180 | >0.05 |
| **De Ferran et al, 2011 [9]** | 0.07±0.97 SDS  (–0.43-0.12) | 0.52±0.79 SDS  (0.12-0.92) | 16 | 0.16 |
| **Oron et al, 2012 [10]** | 163.2±5.8 cm  (161.9-164.5) | 161.7±5.8 cm  (160.4-163) | 73 | 0.12 |
| **Liimatta et al, 2018 [11]** | 167.2 cm  (164.8-169.6) | 166.5 cm | 30 | 0.06 |
| **Riberiro et al, 2019 [12]** | 162.8±7.3 cm  (151-179) | NA | 34 | NA |
| **Mejorado-Molano et al, 2022 [4]** | 164.60 cm  (163.36-165.85) | 163.56 cm  (162.39-164.73) | 79 | 0.06 |
| **This study** | 161.7±6.37  (148.8-175.2) | 160.2±5.42  (149.5-170.9) | 75 | 0.12 |

* Data not available.
